# Lateral ventricle volume trajectories predict response inhibition in older age—A longitudinal brain imaging and machine learning approach

**DOI:** 10.1371/journal.pone.0207967

**Published:** 2019-04-02

**Authors:** Astri J. Lundervold, Alexandra Vik, Arvid Lundervold

**Affiliations:** 1 Department of Biological and Medical Psychology University of Bergen, Norway; 2 Mohn Medical Imaging and Visualization Centre, Department of Biomedicine, University of Bergen, Norway; University of Zurich, SWITZERLAND

## Abstract

**Objective:**

In a three-wave 6 yrs longitudinal study we investigated if the expansion of lateral ventricle (LV) volumes (regarded as a proxy for brain tissue loss) predicts third wave performance on a test of response inhibition (RI).

**Participants and methods:**

Trajectories of left and right lateral ventricle volumes across the three waves were quantified using the longitudinal stream in Freesurfer. All participants (*N* = 74;48 females;mean age 66.0 yrs at the third wave) performed the Color-Word Interference Test (CWIT). Response time on the third condition of CWIT, divided into fast, medium and slow, was used as outcome measure in a machine learning framework. Initially, we performed a linear mixed-effect (LME) analysis to describe subject-specific trajectories of the left and right LV volumes (LVV). These features were input to a multinomial logistic regression classification procedure, predicting individual belongings to one of the three RI classes. To obtain results that might generalize, we evaluated the significance of a *k*-fold cross-validated f1-score with a permutation test, providing a *p*-value that approximates the probability that the score would be obtained by chance. We also calculated a corresponding confusion matrix.

**Results:**

The LME-model showed an annual ∼ 3.0% LVV increase. Evaluation of a cross-validated score using 500 permutations gave an f1-score of 0.462 that was above chance level (*p* = 0.014). 56% of the fast performers were successfully classified. All these were females, and typically older than 65 yrs at inclusion. For the true slow performers, those being correctly classified had higher LVVs than those being misclassified, and their ages at inclusion were also higher.

**Conclusion:**

Major contributions were: (i) a longitudinal design, (ii) advanced brain imaging and segmentation procedures with longitudinal data analysis, and (iii) a data driven machine learning approach including cross-validation and permutation testing to predict behaviour, solely from the individual’s brain “signatures” (LVV trajectories).

## 1 Introduction

Normal aging is associated with morphometric changes in several brain regions and changes affecting cognitive function. The trajectories of age-related changes are, however, characterized by a large interindividual heterogeneity [1]. This is observed in studies of structural brain changes [2], the rate and extent of cognitive changes [3, 4] as well as brain-cognition relations in older age [5, 6], leaving some individuals with preserved cognitive function into old age, and others with a decline at a much younger age. In the severe end of the distribution, the most extensive tissue loss is associated with dementia, a syndrome defined by a severe decline in cognitive function [7]. On the other end of the scale we find so-called “superagers” [8]. They show maintained cognitive function into old age [9], with a corresponding preservation of brain structure over time [6, 10]. This heterogeneity can be explained by several biological and genetic factors, as well as the many life-events and life-style factors that influence an individual through his or her life-time [11–13]. It has for example been shown that compensatory strategies developed through the life-time can slow down a cognitive decline in spite of a decline at a neuronal level [4]. This large number of unknown factors gives arguments for the relevance of a data driven approach when we investigate the relation between subject-specific structural brain changes and cognitive function in the present study.

Several previous studies have related changes in cognitive function to changes in specific regions and structures of the brain (e.g., [14–17]). For example, prefrontal cortex has been linked to global aspects of cognitive function like fluent intelligence [18] and to specific measures defined within the concept of executive function (e.g., [19, 20]). Executive function (EF) is of special interest in studies including older participants, as EF has been described as a hallmark of cognitive aging [21, 22]. In the present study we have focused on response inhibition (RI), which is described as one of the core functional subcomponents of EF [23], susceptible to impairment as part of normal cognitive aging [24, 25]. The close relation between RI and fluent intelligence [26] and between fluid intelligence and various properties of brain structure [18] add to the interest of this EF subcomponent in relation to brain changes. The nature and empirical specificity of such relations between brain regions and RI is, however, still not clear. Inconsistent results are reported and can at least partly be explained by individual differences in age-related volume changes across different brain regions [27], but also by what Salthouse et al. [26] refer to as the “ability impurity” of EF tests. In fact, subfunctions of EF are most likely dependent on multiple, interconnected brain regions [23]. In the present study, we will therefore not use volume changes in specific brain tissue regions or structures as predictors of RI, but rather use trajectories of change in the lateral ventricle (LV) volumes as a proxy of age-related brain tissue loss. This because the lateral ventricular volumes (LVV) can be seen as a “complement volume” of brain parenchyma since the intracranial volume (ICV) is regarded constant during adulthood and older age.

The choice of LV volumes (LVV) is further supported by studies describing the brain’s fluid-filled ventricles as a biomarker of the aging brain [28, 29], and studies linking age-related ventricular expansion to changes in cognitive function at a subject-specific level [30–32]. A study by Todd et al. [33] showed a strong linear relationship between LVV expansion and worsening of cognitive performance over a two-years period. The study assessed cognitive function by tests primarily designed to reveal symptoms of major neurocognitive disorders. Less is known about the longitudinal relationship between LVV expansion and more specified measures of cognitive functions that are prone to normal age-related changes. The eight year longitudinal study by Leong et al. [27] is an exception. The study assessed the co-evolution of volumetric brain changes and cognitive function in a large group of healthy older adults (n = 111, age range 56-83 yrs at baseline) including test measures defined within specified cognitive domains. The results showed volumetric reduction of tissue across several brain regions, and that faster cerebral atrophy and ventricular expansion (at 3.56%/year) were associated with rapid decline in performance on tests of verbal memory and executive function.

The studies referred to above motivated us to further investigate the ability of predicting RI from LVV-derived biomarkers. Response inhibition is here defined from performance on the third condition of the Color-Word interference (CWIT) test, which is part of the Delis and Kaplan Executive Function Scale (D-KEFS) [34]. Previous studies have controlled for the first two conditions of CWIT (color naming and word reading) in a linear regression model to obtain a more “pure” measure of inhibition [25, 35]. In the present study we rather consider the complexity of the third condition as a strength, because it potentially gives a better match to the selected “global” measure of tissue loss (i.e. LVV changes) and is also easier to interpret (RT in seconds). Segmentation of the longitudinal 3D T1-weighted MRI recordings were used to measure the subject-specific trajectories of LVV change across the three study waves, and the RI performance at the third wave was included as an outcome variable, assuming that neuronal loss tends to precede cognitive decline in older age [4].

We see the application of (i) a longitudinal design, (ii) advanced brain imaging and segmentation procedures with longitudinal data analysis (LDA), and (iii) a data driven machine learning approach including cross-validation and permutation testing to predict behavior as the major contributions of the present study. Our aim was to predict RI performance (slow, medium, fast) solely from the individual’s brain “signatures” in terms of LVV trajectories, i.e. expressing and testing subject-specific brain-behavior relationships. By this, we wanted to contribute with methods and results that are likely more generalizable to unseen data than those obtained using ordinary linear regression or classification models applied to the full cohort without using hold-out or a train-test-split cross-validation procedure. More specifically, after image segmentation we used a linear mixed-effect (LME) analysis similar to Leong et al. [27] to describe and select characteristics of the subject-specific LVV trajectories of the left and right lateral ventricle. From explorative data analysis, four features derived from the random effects component in the LME model were included in a multinomial logistic regression classification procedure, predicting individual belongings to one of three classes of performance level (slow, medium and fast) on the RI test. A permutation test was used to evaluate the significance of a cross-validated F1-score to obtain results that may generalize to other samples (i.e. providing a *p*-value that approximates the probability that the score would be obtained by chance). From cross-validation, single subject predictions were obtained, enabling computation of a confusion matrix for better assessment and interpretation of our classifier performance.

From this, we expected to confirm the volume expansion profiles of the lateral ventricles that Leong et al. [27] reported from their statistical mixed effects model, as well as an association between LVV expansion and RI performance.

In the explorative data analysis we expected to reveal an age-related expansion of the lateral ventricle volumes [27], a slower age-related expansion of LVV in females than in males [36, 37], and that poor response inhibition performance is a more frequent in older age [24, 25]. By casting our brain and behavior measurements into a comprehensive classification framework, we hypothesized that model-based features characterizing the LVV trajectories of an individual could act as predictors of *his or her* RI performance. According to previous studies (see e.g. [1]), the success-rate of this prediction was expected to scale with age, with better classification performance in the oldest segment of our cohort.

## 2 Methods

### 2.1 Sample

The study included a cohort of 74 healthy middle-aged and older subjects (48 females and 26 males). They were all part of a three-wave longitudinal study on cognitive aging, where subjects with a history of substance abuse, present neurological or psychiatric disorder, or other significant medical conditions were excluded from participation (see [38, 39] for more details). Their mean age was 59.9 yrs (SD 7.3), 63.3 yrs (7.2) and 66.0 yrs (7.2) for study wave 1, 2 and 3, respectively, and their mean education was 13.94 yrs (2.9). All the 74 subjects provided MRI data across the three study-waves that could be successfully processed during cross-sectional Freesurfer segmentation without need of (subjective) manual editing, and were then run through the longitudinal stream of Freesurfer [40] (details in Section 2.3). Results from the CWIT cognitive test of RI, administered as part of the third study-wave, were available for all the 74 subjects. With an aim to investigate the opportunity and success of predicting performance on a cognitive test from individual trajectories of volumetric brain measures, we decided to restrict the sample derived from our larger study of cognitive aging to those 74 with a complete brain-cognition data set across the three waves.

An inspection of the neuropsychological test data from the three waves confirmed that none of the participants showed results indicating dementia. The test battery included two subtests from the Wechsler Abbreviated Scale of Intelligence (WASI, [41]) administered in the first wave to estimate intellectual function, and the Mini Mental Status Examination (MMSE, [42]) in waves 2 and 3. All participants obtained a MMSE score ≥ 25, and their mean IQ score was 117.1 (sd = 10.2). None of the participants reported or obtained a score on the second edition of the Beck Depression scale (BDI-II) [43] that indicated depression.

All participants signed an informed written consent form, and the study was approved by the Regional Committees for Medical and Health Research Ethics of Southern (study wave 1) and Western Norway (study wave 2 and 3).

### 2.2 Response inhibition

The total raw response-time (RT) score (in seconds) for correct responses on the third condition of the CWIT [34], performed as part of the third study wave, was included as the measure of RI. In this condition, subjects are requested to name the colors of color-words printed in incongruent colors (e.g., the the word “red” printed in “green”) as fast and correct as possible. From this, it is assumed that the participant has to inhibit the more automatic response to read the word, commonly referred to as the Stroop effect. In the two preceding conditions of CWIT, the participants named a set of colours and read a set of color words. The third condition thus includes the effects of these two fundamental abilities [35]. Trained research assistants administrated the test in a quiet room designed for a neuropsychological examination.

### 2.3 MRI acquisition and brain segmentation

Multi-modal MR imaging was performed on a 1.5 T GE Signa Echospeed scanner (MR laboratory, Haraldsplass Deaconess Hospital, Bergen) using a standard 8-channel head coil. Two consecutive T1-weighted 3D volumes were recorded from each subject (to improve SNR and brain segmentation) using a fast spoiled gradient echo (FSPGR) sequence (TE = 1.77 ms; TR = 9.12 ms; TI = 450 ms; FA = 7°; FoV = 240 × 240 mm^2^, image matrix = 256 × 256 × 124; voxel resolution = 0.94 × 0.94 × 1.40 mm^3^; TA = 6:38 min). The same scanner (no upgrades) and T1-w 3D imaging protocol were used at each of the three study waves.

**Fig 1:**
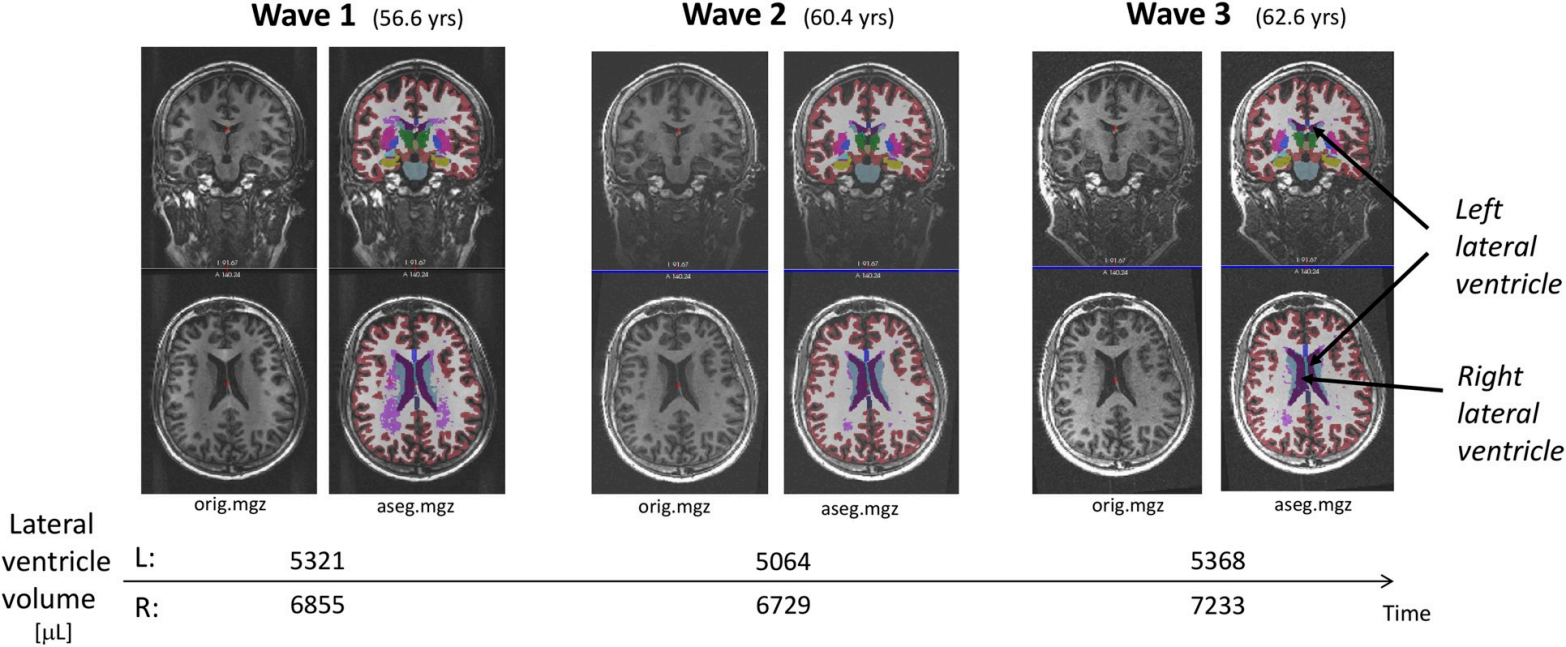
The longitudinal MRI recordings (orig.mgz) and the corresponding Freesurfer segmentations (aseg.mgz) from one of the participants at each of the three study waves. The age at the MRI examinations and corresponding left and right lateral ventricle volumes are given along the time-line.

Brain segmentation and morphometric analysis across the three waves was conducted using the Freesurfer image analysis suite, version 5.3 (documented and freely available online from https://surfer.nmr.mgh.harvard.edu). To extract reliable volume estimates and their trajectories (e.g. left and right lateral ventricles), the cross-sectionally processed images from the three study waves were subsequently run through the longitudinal stream [44] in Freesurfer. Specifically, an unbiased within-subject template space and image is created using robust, inverse consistent registration [45]. Several processing steps, such as skull stripping, Talairach transforms, atlas registration as well as spherical surface maps and parcellations are then initialized with common information from the within-subject template, significantly increasing reliability and statistical power [44]. As a consequence of the longitudinal processing stream and within-subject registration, the estimated total intracranial volume (eTIV) for a given subject remains fixed across the three study waves. To illustrate data, processing stream, and results Fig 1 depicts the longitudinal MRI original recordings (orig.mgz) and the corresponding Freesurfer segmentations (aseg.mgz) from one randomly selected participant at each of the three study waves. The age at the MRI examinations and corresponding left and right lateral ventricle volumes are shown along the time-line.

After running Freesurfer to its end on the collection of subjects, cross sectionally and followed by the longitudinal stream (several days on a standard Linux workstation), we obtained for each wave subject-specific Freesufer directories containing segmentation results (e.g. aseg.mgz for inspection) and aggregated morphometric statistics (e.g. volume of left and right lateral ventricle and the intracranial volume, eTIV being constant for each subject, all in microliter). It was then easy to extract the volumetric data in tabular form for the whole cohort using a Python script. The subject’s age at MRI examinations wave 1, 2 and 3 was derived from the 3D T1-w DICOM headers. We further combined these variables with subject gender and RI reaction time at wave 3 to a single data frame, that also included the eTIV-normalized lateral ventricle volumes, $\frac{\text{LVV}}{\text{eTIV}}$. This Pandas data frame was used in the following analyses.

### 2.4 Statistical analyses

#### 2.4.1 Identification of individual trajectories of LV volume changes

Mixed effects modelling was used to characterize individual trajectories of LVV change according to the following LME model equation:

$$Vol_{ij}^{H}=\beta_{0}^{H}+\beta_{1}^{H}Age_{ij}+\left(b_{0i}^{H}+b_{1i}^{H}Age_{ij}\right)+\epsilon_{ij}^{H},$$

where *H* ∈ {*L*, *R*} denote hemisphere, *i* is subject (*i* = 1, …, *N* = 74) and *j* is wave (*j* = 1, …, *n* = 3). The response variable *Vol*_*ij*_ is volume of left (or right) lateral ventricle in subject *i* at wave *j*, and *Age*_*ij*_ (predictor) is age [years] of subject *i* at wave *j*. The variables *β*_0_ and *β*_1_ are fixed effects model parameters, *b*_0*i*_ and *b*_1*i*_ are random effects model parameters, and *ϵ*_*ij*_ is random residual errors, with zero mean and constant variance *δ* = *ϵ*2.

**Fig 2:**
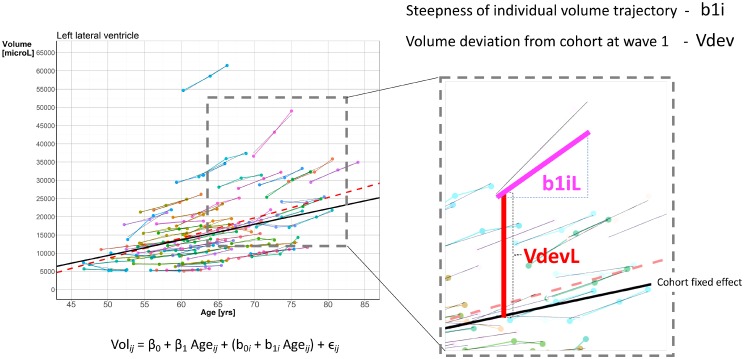
Illustration (left hemisphere) of the subject-specific measures (b1iL, b1iR, VdevL, VdevR) of LVV trajectories obtained from the LME analysis.

Two features were derived from the LME model to characterize the individual LVV trajectories. The first (denoted b1i) describes the steepness of individual volume trajectory, defined as the slope parameter in a two-parameter family of random effects (*b*_0*i*_, *b*_1*i*_). The second feature (denoted Vdev) describes an LVV deviation measure at baseline, and is defined as the difference at wave 1 between subject-specific LVV and the age-matched LVV expected from the cohort fixed effect regression line that is parameterized with (*β*_0_, *β*_1_). For each of these features, one is selected from the right and one from the left hemisphere. This is motivated from expected similar, but not necessarily identical patterns of LVV trajectories in the left and the right hemisphere, and also possible hemispheric differences as reported in previous studies (e.g. [46]). These four model-based features (b1iL, b1iR, VdevL, VdevR) were included as predictors in the further analyses (see Fig 2 for illustration).

#### 2.4.2 Explorative data analysis

The distributions (i.e. kernel density estimation) and Pearson correlations between the six parameters: age at wave 3 (Age3), the four LVV measures (b1iL, b1iR, VdevL, VdevR), and the reaction time from the RI measure at wave 3 (RI3) were calculated and presented separately for females and males as a comprehensive generalized pairs plot using the ggplot2 and GGally packages in **R** ver 3.5.

#### 2.4.3 Prediction of response inhibition

A classification approach with three categories of RI performance was used to investigate the predictive value of the four LVV measures. To generate such categories, the participants were divided into slow, medium, and fast performers. First, a jittering procedure was used to eliminate RT ties, adding Gaussian $N(\mu =0,\sigma^{2})$ noise with *σ* = 0.05 to the integer valued reaction times, being in the range [35, 102] (in seconds), such that each jittered RT was typically around ±50 ms from the measured one. A quantile-based discretization function was then used to compute four reaction time threshold values and corresponding reaction time intervals to obtain balanced classes, i.e. close to the same number of participants in each category (cf. Table 1).

**Table 1:** Definitions and characteristics of fast, medium, and slow performers.

RI label	RT interval [sec]	F	M	Total
fast	[34.9, 49.9〉	19	6	25
medium	[49.9, 58.7〉	14	10	24
slow	[58.7, 102.1〉	15	10	25

RI = response inhibition; RT = reaction time; F = number of females; M = number of males.

For predicting category *y*_*i*_ ∈ {slow, medium, fast} from explanatory variables *X*_*i*_ = (b1iL_*i*_, b1iR_*i*_, VdevL_*i*_, VdevR_*i*_) where *i* ∈ {1, …, 74} denote participant number *i*, we used a linear regularized logistic regression classifier as implemented in Logistic Regression from the linear models in the scikit-learn library for Python. Since we have a three-class problem, we used a multinomial version with the cross-entropy loss, a limited-memory Broyden—Fletcher—Goldfarb—Shanno (‘lbfgs’) solver, L2 regularization with primal formulation, tolerance for stopping criteria 0.0001, and let 500 be the maximum number of iterations taken for the solver to converge. We fixed the value of parameter *C* (the inverse of regularization strength in the algorithm) to be 0.5 in all our classification experiments without any hyperparameter tuning.

The best and most detailed description of the classifier being used is found in https://scikit-learn.org/stable/modules/generated/sklearn.linear_model.LogisticRegression.html and the references therein.

If a feature has a mean and variance that is orders of magnitude larger than others, it might dominate the objective function (cross-entropy loss) and make our classifier using L2 regularization unable to learn from other features correctly, as expected. Such effect could be observed by assessing feature importance before and after preprocessing with a scaler (mean removal and variance scaling). To assure that all features were centered around 0 and have variance in the same order, the input data were preprocessed with scikit-learn’s StandardScaler obtaining zero mean and unit variance for each feature, also in every fold during cross validation (see below).

#### 2.4.4 Evaluation using *k*-fold cross validation with permutations

It is well known that learning the parameters of a prediction function and testing it on the same data is a methodological mistake. A sufficiently expressive model would just repeat the labels of the samples that it has just seen and could have a perfect score but fail to predict anything useful on yet unseen data, being a victim of *overfitting* and lack of *generalization abilities*. When performing our supervised machine learning experiments on labeled data (we let our complete dataset be denoted (*X*, *y*) where *X* is the 74 × 4 matrix of predictors and *y* is the 74 × 1 vector of corresponding RI labels), a common practice is therefore to hold out part of the available data as a training set used for model estimation and the remaining samples as a test set for performance evaluation. However, by partitioning the available data into two sets (or, three when including a validation set for hyperparameter tuning), we drastically reduce the number of samples which can be used for learning the model, and the results can depend on a particular random choice for the pair of train and test datasets. To ameliorate this problem, especially in small-sample size studies like ours, we used *k*-fold cross-validation (CV) to assess the prediction properties of our multinomial logistic regression classifier, such as performance scores (i.e. accuracy, precision, recall, and f1) and confusion matrices. In this procedure the dataset was split into *k* smaller sets (stratified folds were made by preserving the percentage of samples for each class), and for each of the *k* folds, a model was trained using *k* − 1 of the folds as training data, and the estimated model was then applied on the remaining fold being used as a test dataset to compute performance scores. The performance measure reported by *k*-fold cross-validation was the average of the values computed in the loop. In our analysis we report on average (‘micro’) f1-score, calculated globally by counting the total true positives (*TP*), false negatives (*FN*) and false positives (*FP*), and interpreted as a weighted average of the precision = *TP*/(*TP*+ *FP*) and the recall = *TP*/(*TP* + *FN*), i.e. *f*1 = 2(precision × recall)/(precision + recall).

In order to test if a classification score was significant, a technique of repeating several times the *k*-fold CV classification procedure after randomizing the labels was used, i.e. evaluating the significance of a cross-validated score with permutation testing. By this means, a *p*-value approximates the probability that the score would be obtained by chance is given by the percentage of runs for which the score obtained was greater than the classification score obtained in the first place. In our experiments we used the permutation_test_score function in scikit-learn with *k* = 5, and 500 permutations, yielding a *p*-value = (*C* + 1)/(500 + 1), where *C* is the number of permutations whose score ≥ the true score. The minimum *p*-value is 1/(500 + 1) ≈ 0.002 corresponding to the case where the classifier is so good that none of the classifiers with shuffled labels has a better score, and the worst value is 1.0. The permutation_test_score computations returned the true score without permuting labels, an array of scores for each permutation, and the *p*-value described above. These are reported in the Results section. To further assess our model, we generated cross-validated estimates for each of the 74 data points in *X* (with corresponding RI label *y*) using the same *k*-fold cross-validation and standard scaling as described above. Mapping each data point in the input to the prediction that was obtained for that element when it was in the test set, was done for diagnostic purposes—illustrating typical confusion matrices and scores obtained from the model—not for measuring generalization error as was previously done in the permutation testing. Finally, we computed the 3 × 3 *confusion matrix* using the true labels versus the classification labels returned from the cross validation prediction.

**Fig 3:**
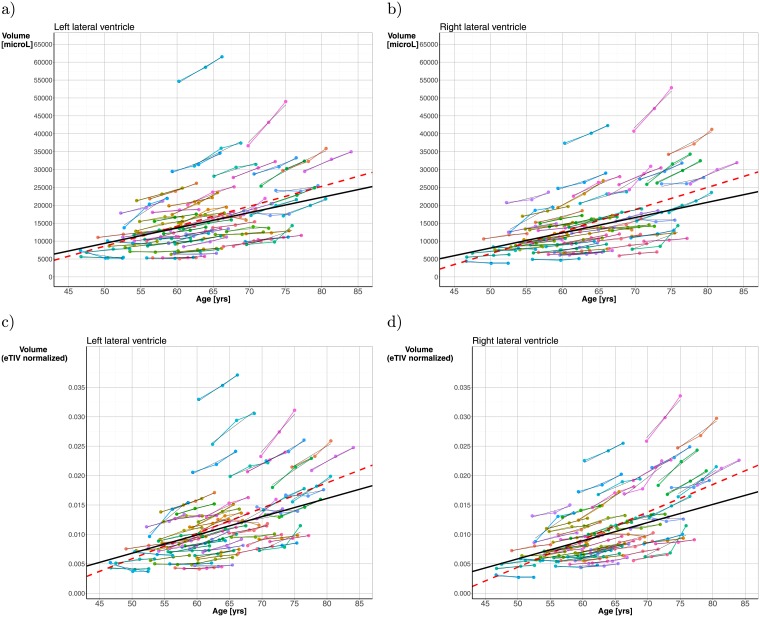
Subject-specific longitudinal lateral ventricle volumes versus age in left (**a**) and right (**b**) hemisphere shown as color-coded spaghetti plots across the three study waves. For left and right hemisphere the random effects, estimated from the linear mixed-effect model *Vol*_*ij*_ = *β*_0_ + *β*_1_ *Age*_*ij*_ + (*b*_0*i*_ + *b*_1*i*_ *Age*_*ij*_) + *ϵ*_*ij*_, are depicted as thin line segments in black superimposed on the color-coded line plots. The thick regression line in black represents the estimated fixed effect, and the broken line represents ordinary linear least squares regression (OLS) line. Subject-specific longitudinal *eTIV-normalized* lateral ventricle volumes versus age in left (**c**) and right (**d**) hemisphere, respectively, are shown as color-coded spaghetti plots across the three study waves. Here, a linear mixed-effect model was applied and fitted to the eTIV-normalized data.

**Fig 4:**
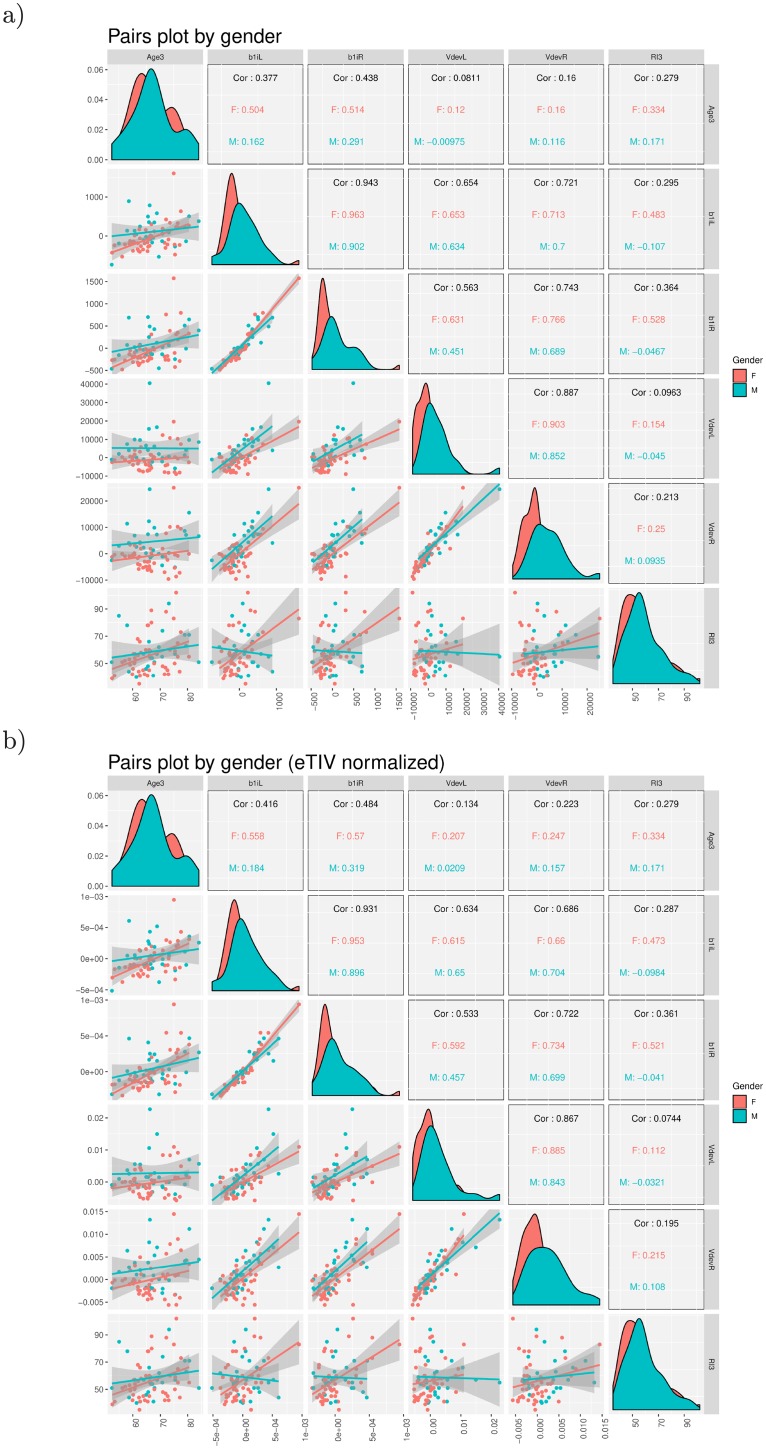
Generalized pairs plot depicting the kernel density estimated empirical distributions of each of the six variables and Pearson correlations between age, the four LVV trajectory measures and response inhibition. (a) Non-normalized LVVs. (b) eTIV-normalized LVVs. The graphs and correlations are given separately for females (in red) and males (in green). Age3 = age of participant at study wave 3; b1iL = LVV steepness measure, left hemisphere; b1iR = LVV steepness measure, right hemisphere; VdevL = LVV deviance measure, left hemisphere; VdevR = LVV deviance measure, right hemisphere; RI3 = response inhibition reaction time at study wave 3.

All analyses were implemented as Jupyter notebooks using Python (3.6), Numpy (1.14), Pandas (0.23), Matplotlib (3.0), Statsmodels (0.9), Scikit-learn (0.20), and rpy2 (2.9) with R (3.5) and packages lme4, ggplot2 and GGally for producing Figs 3, 4 and 6. These notebooks with corresponding datasets as .csv files were tested to run under Anaconda on both MacOS 10.14, Windows 10, and Ubuntu 18.04 platforms and will be available on GitHub [https://github.com/arvidl/lvv-ri].

## 3 Results

### 3.1 Three wave changes in lateral ventricular volumes

From the linear mixed-effect (LME) model used to investigate the age-related evolution of the ventricular volumes Fig 3 shows the fixed (fat unbroken line) and random effects (thin line segments) calculated from the LVV modeling for the left (a) and right (b) hemispheres, and the corresponding data and LME-model fitted to the eTIV-normalized LVV values (left (c) and right (d) hemispheres). The fixed effects regression line shows expansion of LV volumes (or, eTIV-normalized LVVs) with increasing age. From the fixed effect model we found an overall cohort volume increase of 429 *μ*L/year for the left side LVV, and 426 *μ*L/year for the right side. With a mean LVV in left hemisphere of 14994 [*μ*L] at inclusion, this represents an annual ≈ 2.9% increase in left side LVV, and with a mean LVV in right hemisphere of 13777 [*μ*L], this represents an annual ≈ 3.1% right side LVV increase. Visual inspection reveals a trend towards a steeper slope for the older participants in the cohort. Furthermore, the fixed effects regression line was less steep than ordinary linear least squares regression line (fat broken line), demonstrating the effect of the LDA approach that takes into account the *dependencies* between the subject-specific measures across the three study waves.

### 3.2 Explorative data analysis

Fig 4 shows the kernel density estimated distributions of age, the four volume measures, and the response inhibition performance (RI), and their pair-wise Pearson correlations, with separate panels for the use of non-normalized LVVs with respect to the subject’s ICV (a), and the eTIV-normalized LVVs (b).

**Fig 5:**
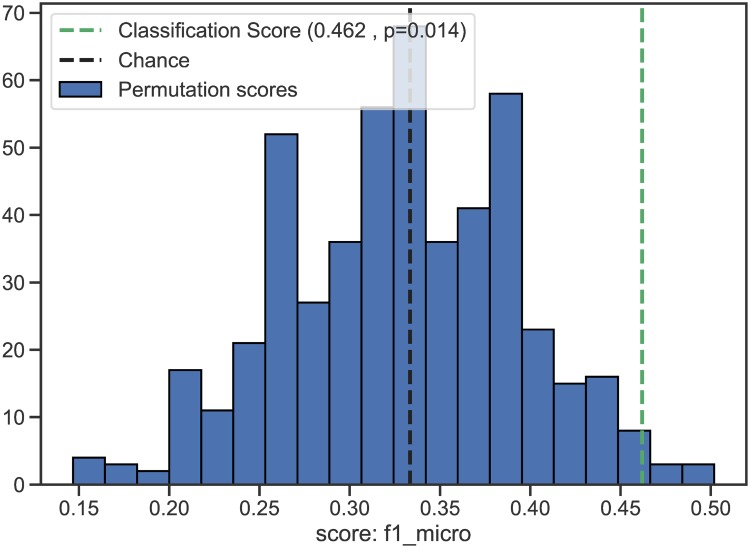
Result from the simulation experiments assessing the significance of a 5-fold cross-validated score (f1) with 500 permutations using multinomial logistic regression. The predictors are *X* = {b1iL, b1iR, VdevL, VdevR} and the classes are the three levels of RI reaction times, *y* = {slow, medium, fast}.

The gender effects are shown by presenting the results separately for females (*n* = 48) and males (*n* = 26). The LVV-derived measures for females were shifted towards the lower end of the distribution compared to males, while the gender-specific distributions were less different for age and RI in both when using native LVVs and eTIV-normalized LVVs. The Pearson correlations were strong between the left (b1iL) and right (b1iR) slope measures in (a) *r* = 0.94 (and also for the eTIV-normalized LVVs *r* = 0.93 in (b)), and between the two deviance measures VdevL and VdevR: *r* = 0.89 in (a), *r* = 0.87 in (b). Statistically significant correlation was found, for females only, between RI3 and b1iL (*r* = 0.48) and between RI3 and b1iR (*r* = 0.53). For the eTIV-normalized LVVs similar correlations were found (in females only). Age at wave 3 was moderately correlated with the four lateral ventricular features in females. In males these correlations were generally lower and non-significant. This was the case for both native LVVs and for eTIV-normalized LVVs. Due to the small qualitative difference between the use of native LV volumes and eTIV-normalized volumes observed in the exploratory data analyses (cf. Figs 4 and 5), we performed our machine learning classification experiments using features derived from the native LV volumes, only.

### 3.3 Predicting response-inhibition from LVV trajectories

The four LME-based features selected to characterize the non-normalized LVV trajectories, i.e. slope of LVV change (b1i) and the LVV deviation at the time of inclusion (Vdev), from both the right and from the left hemispheres, were used to compute our cross-validated score to predict level of RI. Fig 5 shows the results from our simulation experiments using iteratively fitted multinominal logistics regression models (*n* = 500 permutations) to assess the significance of the f1-score. The vertical green dotted line represent our cross-validation classification score of 0.462 and shows that the score is significantly better (*p* = 0.014) than the 0.333 chance level (black dotted line).

The results from the *k*-fold cross-validation procedure is presented in Table 2. The precision (positive predictive value) is higher than the recall (sensitivity) for the slow and medium RI classes, but lower than the recall score for fast performers. The overall slightly best f1-score was obtained for the fast performers. The fast performers also had a recall score that was higher than any other score metric, regardless level of performance.

**Table 2:** Predictions from each split of cross-validation, generating cross-validated estimates for each input data point using multinomial logistic regression.

	f1-score	precision	recall	support
slow	0.4255	0.4545	0.4000	25
medium	0.4545	0.5000	0.4167	24
fast	0.4912	0.4375	0.5600	25
*micro avg*	0.4595	0.4595	0.4595	74
*macro avg*	0.4571	0.4640	0.4589	74
*weighted avg*	0.4571	0.4635	0.4595	74

f1-measure, precision, recall, and support for each class are computed. The predictors are *X* = {b1iL, b1iR, VdevL, VdevR} and the classes are the three levels of RI reaction times, *y* = {slow, medium, fast}. The reported averages include *micro average* (averaging the total true positives, false negatives and false positives), *macro average* (averaging the unweighted mean per label), and *weighted average* (averaging the support-weighted mean per label). The support is the number of occurrences of each class in *y*_true_.

**Fig 6:**
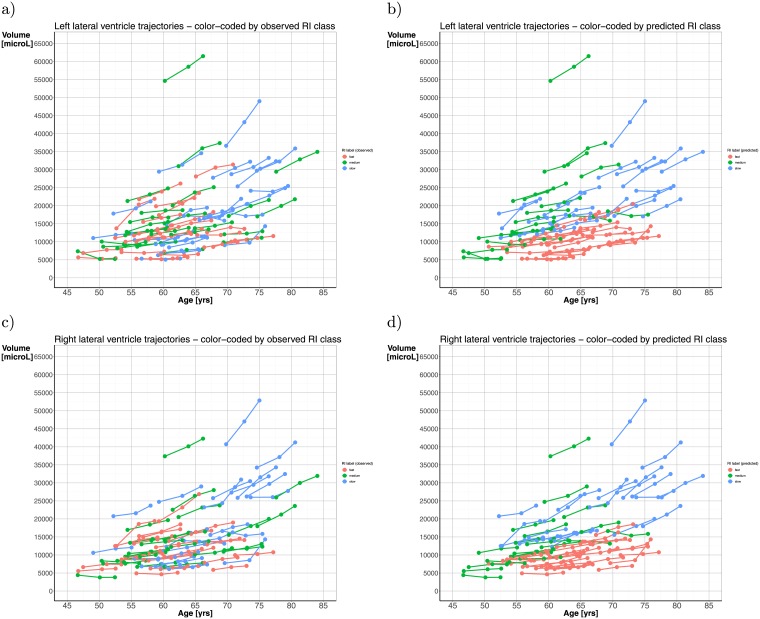
Plots showing the observed RI labels (leftmost two panels, for left (a) and right hemisphere (c), respectively) and the predicted RI labels (rightmost two panels, for left (b) and right hemisphere (d), respectively) for each of the 74 subjects in the cohort. When a given trajectory in (a) or (c) changes its color as it occur in (b) or (d), that subject is misclassified; otherwise he or she is correctly classified with respect to RI performance.

Fig 6 illustrates the 74 subject-specific trajectories color-coded with the **observed (true) RI label** for left hemisphere (a) and the right hemisphere (c). The same 74 subject-specific trajectories are then color-coded with the **predicted RI label** for left hemisphere (b) and the right hemisphere (d). The most successful classification, in both hemispheres, is for the fast performers as illustrated by the red line-segments. The slow performers, shown by the blue line-segments, seem to be most successfully classified if their age was above 65 years at inclusion.

**Fig 7:**
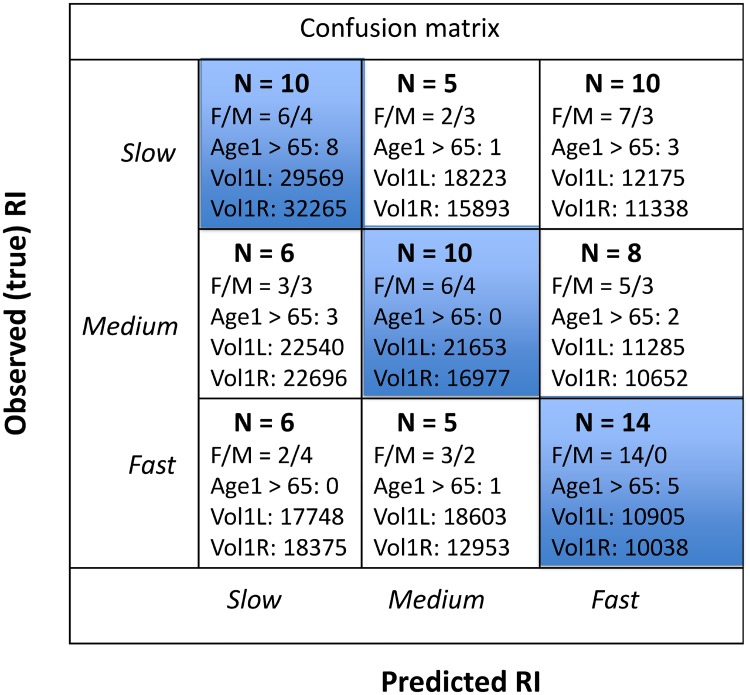
The 3 × 3 *confusion matrix* computed for the slow, medium and fast RI labels returned from the cross validation prediction with our multinomial logistic regression model compared with the co-occurrences of the true (observed) RI labels. The diagonal cells are those representing correctly classified subjects (number of occurrences in each cells are given as *N*), and these cells are shaded in blue. Off-diagonal cells represents various events of misclassification. Observed/predicted co-occurrences are also accompanied, for each cell, with corresponding information about gender ratio (F/M), confirmed age at inclusion larger than 65 years (Age1 > 65), and volume means in microliters of left and right lateral ventricle (Vol1L and Vol1R), respectively, at time of subject inclusion in the study.

The 3 × 3 *confusion matrix* (CM) compares the true labels (Observed RI in Fig 7) versus the classification labels returned from the cross validation prediction (Predicted RI in Fig 7). We have also computed CM cell-specific information about gender ratio (F/M), number of participants older than 65 years at baseline (Age1 > 65), and volume means in microliters of left and right lateral ventricle (Vol1L and Vol1R), respectively, at baseline. The confusion matrix in Fig 7 shows that $\frac{14}{25}=56\%$ of the fast performers were correctly classified, all were females, and five of these were older than 65 years at inclusion. Only one participant older than 65 at inclusion who was a fast performer was misclassified. We also found that the correctly classified fast performers were among those who had the smallest LVVs at baseline. The fast performers who were misclassified also had larger LVVs at inclusion. For the true slow performers, those being correctly classified had higher LVVs than those being misclassified, and their age at inclusion were also higher. A relatively high proportion (40%) of the slow performers were misclassified as fast. In this group there were more females than males, few were older than 65 years at inclusion, and their LVVs were substantially lower (< 50%) than those being correctly classified.

## 4 Discussion

The present study used an LME model to describe, visualize, and design four features characterizing subject-specific LVV trajectories: slope of his or her volume change across the three study waves and a measure of age-related deviance between cohort LVV and subject LVV at inclusion in the study. These LME-based features where then input as predictors of level of RI performance using a linear regularized multinomial logistic regression classifier within a machine learning framework incorporating *k*-fold cross validation and permutation testing. Visual inspection of the LME results revealed an approximately linear age-related expansion of the lateral ventricle volumes over the six years period of observations. The exploratory data analysis showed that distributions of all four LVV features were characterized by gender differences, and that significant correlations between response inhibition performance, age and the LVV slope measure were mostly restricted to the female part of the sample. A cross-validated score predicted performance defined within the three RI classes with a mean classification f1-score that was moderately good (0.462), and clearly better than chance level (*p* < 0.02). A confusion matrix revealed that fast performers were most successfully predicted. Furthermore, the group of successfully classified fast performers included only females, participants with the smallest LVVs at baseline, and all but one fast performer older than 65 at inclusion. For those being successfully classified as slow performers, 67% were older than 65 years at inclusion and their LVVs were higher that those being misclassified within this slow RI class.

The results confirmed that healthy aging is associated with a slight expansion of the lateral ventricular system. This finding further supports arguments for using information about volume of brain’s fluid-filled ventricles as an imaging-derived biomarker in studies of the aging brain [27–33]. Interestingly, the present study estimated an annual fixed-effect increase in LVVs at ≈ 3% in the cohort (slightly larger in right hemisphere compared to the left), close to the ventricular expansion (3.56%/year) reported in the study by Leong et al. [27]. In addition to the LME-modelling approach, our contribution relates to data from *three* study waves being analyzed (Leong et al. reported results from 111 subjects in a two-waves-study), and that we were taking the analysis one step further, bringing the data into a predictive machine learning (ML) framework. By this, we obtained results that could be applied at a single case level, being obtained with a method (*k*-fold cross validation) that are aimed to have generalization abilities and thus being applicable to yet-unseen data. In this ML context we could show that different classes of RI performance (slow, medium and fast) could be predicted from the LVV trajectories with an accuracy and f1-score that was moderate but clearly above chance level, and further emphasize the importance of gender and age illustrated by the explorative data analysis and the extended confusion matrix.

The confusion matrix showed that all fast performers who were correctly classified were females, and that the overall percentage of correctly classified females (54%) was higher than for males (31%). These results demonstrate the importance of gender, which was also shown in the explorative data analysis. Here, the Pearson correlations between level of RI performance and the two LVV slope measures were much stronger in the female part of the sample. By this, our results were similar to the results reported by Aljondi et al. [15] in a female-only sample, using the same Freeurfer longitudinal stream image analysis to obtain atrophy estimates, and a similar linear regression analysis to model brain-cognition changes as in our study. Gender differences in rate of LVV expansion reported in previous studies have indicated a slower expansion in females than males [36, 37]. Results from our explorative data analysis suggested that the rate of expansion is age-related. The slope of the LVV trajectories were lower in females than males in the younger age groups but shifted to a higher value in females in the oldest age groups. The lack of consistent results across studies may thus be related to age differences in the samples. For example, the slower progression of volume expansion in females than males reported by Chung et al. [36] and Hasan and collaborators [37] were based on data from a younger sample than in the present study (i.e. subjects in their 40s and between 18 and 59 years, respectively).

The importance of including participants with age > 65 years was illustrated by the extended confusion matrix being computed in our study. This matrix showed that all but one fast performer who were > 65 at baseline were correctly classified. We may speculate if these fast performers of age > 65, with relatively small LVVs (about 1/3 of LVV for those with slow RI performance), represent what Rogalski and collaborators referred to as “superagers” [8], and that their LVV trajectories can serve (or contribute) as predictors of preserved brain function into old age—at least in females. Future longitudinal studies including a larger sample size, a longer follow-up period and wider age span, are therefore indeed warranted.

We will also emphasize the results obtained from the slow RI performers. Our measures of LVV trajectories correctly classified eight of twelve (67%) slow performers aged > 65 years. If we assume that their slow performance on the RI test reflects a preclinical sign of a Mild Cognitive Impairment (MCI), the results would have important clinical implications. Previous studies have shown that more than 50% of MCI patients are expected to progress and convert to dementia within five years (e.g. [47]). Although speculative, our methods and results may be relevant to efforts in obtaining better and more accurate diagnostic and monitoring tools for brain health in older adults: individual change in the rate of ventricular expansion such as LVVs could act as a sensitive measure of an early stage of a neurodegenerative disease [48].

The somewhat low number of participants (*N* = 74) make us unable to state firm and general conclusions. A larger sample could improve our classifications scheme by incorporating the LME-model estimation within the cross-validation loop, and by this further reduce the risk of “data leakage” (i.e. the training set and the test set sharing information). The value of including of a larger number and diverse set of predictors have been well demonstrated in studies based on theoretical models considering brain maintenance and cognitive reserves (e.g.,[4, 6]. These models emphasize the importance of life-events [4, 49], a richer set of imaging information using multimodal MRI [12, 50] and PET [51, 52]. Together, this provides strong arguments for sharing data (and code) across research groups [53] and use of predictive models and methods within modern machine learning frameworks [50].

### 4.1 Conclusion

We showed that a set of four LME-derived measures of LVV trajectories across three study waves gave a fairly good prediction of RI performance, confirming the role of lateral ventricle volumes as an imaging-based biomarker of cognitive function in older adults. Our major contributions are the application of (i) a three wave longitudinal design, (ii) advanced brain imaging and segmentation procedures with longitudinal data analysis, and (iii) a data driven machine learning approach including cross-validation and permutation testing to predict RI performance solely from the individual’s brain “signatures” (LVV trajectories). Future studies should further investigate this avenue regarding brain-behavior relationships in older age.
